# Consideration for Affects of an XOR in a Random Number Generator Using Ring Oscillators

**DOI:** 10.3390/e23091168

**Published:** 2021-09-05

**Authors:** Ryoichi Sato, Yuta Kodera, Md. Arshad Ali, Takuya Kusaka, Yasuyuki Nogami, Robert H. Morelos-Zaragoza

**Affiliations:** 1 Graduate School of Natural Science and Technology, Okayama University, Okayama 700-8530, Japan; yuta_kodera@okayama-u.ac.jp (Y.K.); kusaka-t@okayama-u.ac.jp (T.K.); yasuyuki.nogami@gmail.com (Y.N.); 2 Department of Computer Science and Engineering, Hajee Mohammad Danesh Science and Technology University (HSTU), Dinajpur 5200, Bangladesh; arshad@hstu.ac.bd; 3 Department of Electrical Engineering, San José State University, One Washington Square, San José, CA 95192, USA; robert.morelos-zaragoza@sjsu.edu

**Keywords:** entropy, field programmable gate array, true random number generator, period, ring oscillator, stomatic process, state transition, XOR gate

## Abstract

A cloud service to offer entropy has been paid much attention to. As one of the entropy sources, a physical random number generator is used as a true random number generator, relying on its irreproducibility. This paper focuses on a physical random number generator using a field-programmable gate array as an entropy source by employing ring oscillator circuits as a representative true random number generator. This paper investigates the effects of an XOR gate in the oscillation circuit by observing the output signal period. It aims to reveal the relationship between inputs and the output through the XOR gate in the target generator. The authors conduct two experiments to consider the relevance. It is confirmed that combining two ring oscillators with an XOR gate increases the complexity of the output cycle. In addition, verification using state transitions showed that the probability of the state transitions was evenly distributed by increasing the number of ring oscillator circuits.

## 1. Introduction

In recent years, a cloud service to offer entropy has been paid much attention to, and such cloud services are called EaaS (Entropy as a Service) [1]. It works as if it is an entropy source through the Internet for various types of devices. This situation would provide a great opportunity for IoT (Internet of Things) devices to obtain an adequate entropy source. It enables us to accelerate the utilization of these poor devices, even for cryptographic purposes toward the Fourth Industrial Revolution (Industry 4.0).

As one of the entropy sources, a physical RNG (random number generator) is used as the source of the TRNG (true random number generator) [2], relying on its irreproducibility. Typically, it is composed of modules that take physical phenomena and convert it into digital (binary) signals. This implicitly means that the circuit size of some types of physical RNGs becomes quite large and not easy to implement for general uses. In this context, there is a stream of research on physical RNGs to ensure that the design has a high entropy and compact hardware size. 

As one of strategies to develop such RNGs, an FPGA (Field Programmable Gate Array) is used for generating a bit stream having high entropy, employed for the source of EaaS. Traditionally, an external noise or human-related motions (e.g., position of mouse pointer) are used as the source of a physical RNG. However, these methods require a different quality of entropy depending on the device, or another source element that provides adequate entropy is needed. Additionally, since the human-related sources always demand humans’ activities, it would not be a better choice for EaaS. In this sense, generators using FPGAs have the advantage that they can yield random numbers independent from the state in the logic circuits and an external connection to a noise source.

There are mainly two candidates for well-known physical RNGs using compact circuits for the authors’ purposes. Though the combinations of those RNGs have been investigated in recent years, the authors begin by reviewing some portion of theoretic properties in this paper to pursue ideal randomness. The first RNG is designed by using an unstable state in a digital logic circuit. The unstable state is called metastability, and it has the advantage of ease of implementation because of its simple structure [3]. Since RNGs using metastability decide on a bit value depending on the stabled output of the RS latch being HIGH (1) or LOW (0), it takes time to generate a bit. On the other hand, a method using ROs (ring oscillators) outputs values by relying on the synchronization of the clock [4,5]. It uses the quantization [6,7] of unstable oscillation signals to give a bit stream.

As related to previous works concerning RNGs using ROs, Sunar et al. have proposed a circuit together with a theoretical viewpoint [8]. Based on their construction, Wold et al. proposed and experimentally showed the construction of an RNG having a better bit distribution together with ROs on FPGA [9]. It is noted that the authors think that the ideal bit distribution is a uniform distribution in the sense that it should be statistically hard to predict the next bit from previous *n*-bits if the output of 0 or 1 is equivalently given by 1/2^n^. One of the common features of these generators is that they use an XOR gate to put the outputs from sources together. These methods are widely used for constructing RO-based RNGs, such as in [10,11,12,13]. However, most of the research omits a discussion concerning the effects of the XOR gate toward the output of the generators. Therefore, this paper motivates one to discuss the reasons why this gate would be required for the generators using the ROs. The strategy for the discussion is to observe and compare the period of the series of the output of the RO and the output of the two ROs when they are combined by an XOR gate.

More precisely, since an RO is a kind of oscillator circuit and has an approximate period, the authors believe a role of the XOR gates is to mix the periods of respective ROs and make the whole period long. It is noted that the RO does not have the exact period of its oscillation due to glitches [5], and its period can be approximately found by observing it over a long span [13]. In this sense, one can say that an RO has an approximate period. In this paper, the authors conduct two types of observations to check the period. First, they plot a random number series on a graph and visually verify its periodicity. Second, they compare and verify the transition probabilities of the random number series by focusing on the probability of the state transitions. We note the relationship between *n*-bits and their following *n*-bits as states of bits in this paper, and state transition means that a state B (e.g., 3-bits 001) appears after a state A (e.g., 3-bits 000).

As a result, it is confirmed that combining two ROs with an XOR gate increases the complexity of the output cycle. In addition, verification using state transitions showed that the probability of state transitions was evenly distributed by increasing the number of RO circuits.

## 2. Preliminaries

This paper focused on a physical RNG using RO circuits on FPGA. This section briefly reviews several fundamental concepts and the basic idea of RO circuits from previous work.

### 2.1. RO Circuit

**Figure 1:**
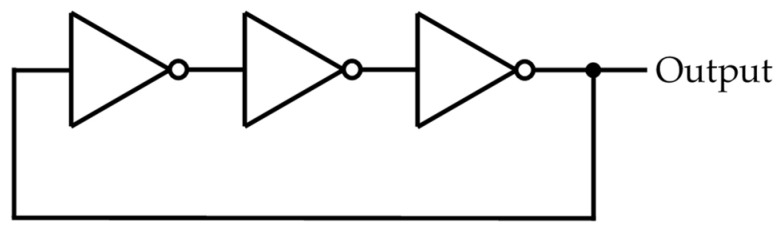
Example of an RO circuit.

One of the typical RO circuits is constructed by an odd number of NOT gates by connecting them in a ring shape, as shown in Figure 1. Then, the output signal of this circuit causes an oscillation, and its approximate period is endowed by the number of NOT gates. In addition, the wire lengths connected to each NOT gate also affect the oscillation period of the RO circuit. 

More precisely, the RO does not have the exact period because the oscillation of an RO circuit vibrates on a time axis affected by some external disturbances such as atmospheric temperature. However, since a glitch can happen in quite a short term rather than the oscillation period, the period can be approximate by ignoring those glitches. 

In this context, the authors simply mention this approximate period as the period of the RO. In particular, the authors focus on and discuss the relationship between the period of the RO and the randomness of available sequences in this paper.

### 2.2. Designs of a Generator in Previous Works

**Figure 2:**
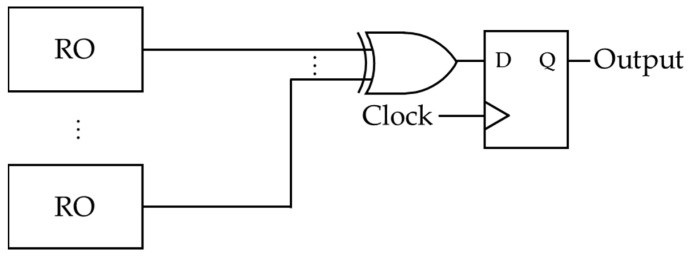
The circuit proposed by Sunar et al.

Several studies on RNG circuits using ROs have been promoted. Sunar et al. proposed a circuit generating a random number sequence using ROs [8], as shown in Figure 2. The circuit is designed to mix the jitter output from each RO by an XOR gate, and the output of the XOR gate is synchronized by an internal clock. In [8], Sunar et al. also showed several theoretical discussions such as a combinational approach and ideal ring lengths. 

**Figure 3:**
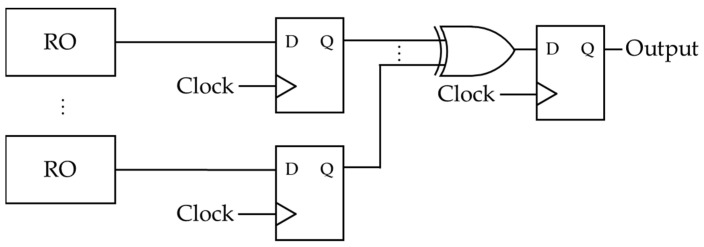
The circuit proposed by Wold et al.

Based on [8], Wold et al. constructed the circuit on FPGA, and they proposed another circuit of an RNG using ROs [9], as shown in Figure 3. The circuit is known to have a better bit distribution than the circuit proposed by Sunar et al. Currently, the construction by Wold et al. is widely adopted. Besides these generators, various physical RNG circuits are designed on FPGAs or CMOS [14]. The readers can get more details by referring to [14], which shows the difference between the random number test method and the construction environment of respective physical RNGs.

In this paper, the authors’ discussion focuses on the latter construction by Wold et al. and investigates the relevance between the periodicity of the ROs and the randomness of the output sequences practically.

### 2.3. The Author’s Target and Implementation

**Figure 4:**
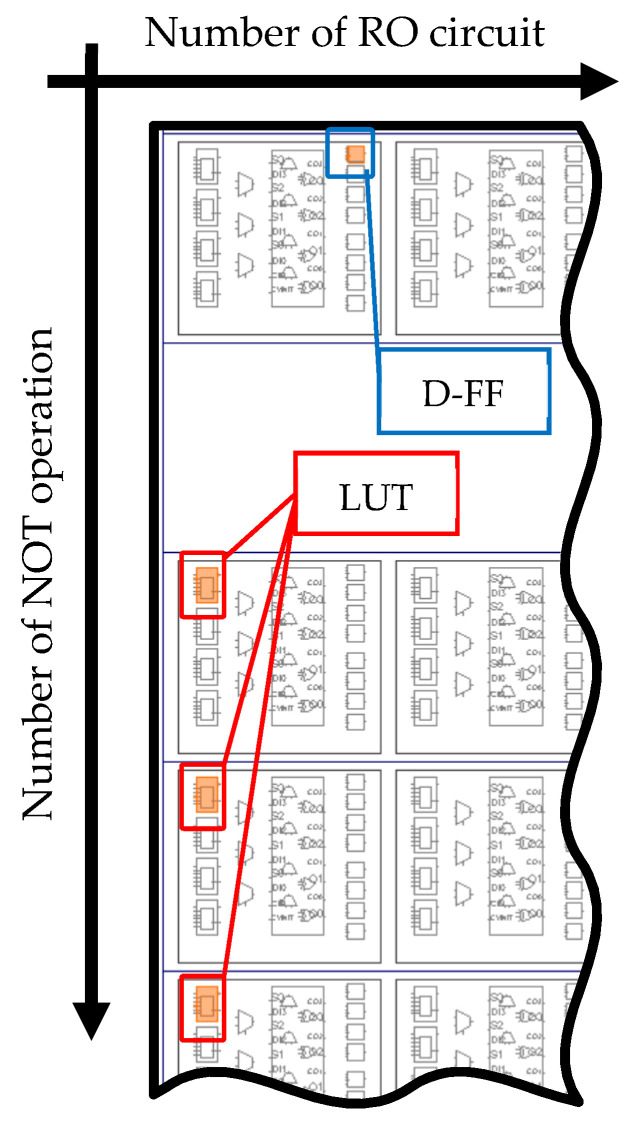
Arrangement of the LUT and the D-FF used in the RO circuit.

This paper mainly deals with the TRNG proposed by Wold et al., as shown in Figure 3, and it is implemented by considering the layout of LUTs (Look-up Tables) and D-FFs (Delay Flip-flops) on an FPGA. Each LUT and D-FF is arranged on FPGA so that the arrangement of each element should be the same for all the RO circuits. This is because the oscillation period of an RO circuit in the generator affects the wire length connected to each LUT and D-FF. The arrangements of the LUT and the D-FF are shown in Figure 4. It is noted that logic gates are expressed by LUTs when one implements a logic circuit on an FPGA. Accordingly, NOT gates and XOR gates used in the physical RNG circuit are also expressed by LUTs in our environment.

More precisely, since some IDEs automatically optimize the arrangement of each element on an FPGA to reduce the redundancy or errors in a circuit, the binary obtained from an IDE is not always as we desired. Therefore, the above options are required, and the following experiments are conducted with them. The following experiments are conducted with the environments shown in Table 1. It is noted that the number of NOT gates in an RO indicates that every RO is composed of three NOT gates, as shown in Figure 1. This aims to observe the effects of an XOR gate when the number of ROs changes.

## 3. Proposals 

In the RNG using RO circuits, the necessity of XOR gates has not been discussed clearly in previous research. Therefore, this section considers its necessity by observing the period of the output sequence endowed by an XOR gate.

### 3.1. Graphical Periodicity Test

The first experimental observation uses a part of a sequence obtained from an RNG on an FPGA. The authors propose to use the *n^2^*-bits length of the sequence to make an *n* × *n* image to visualize the implicit periodicity of the RNG. For convenience, let *n* = 32 in what follows.

The observation is conducted by the following steps:

1. Split the output sequence into 32-bit words without any duplications and regard them as the row vector of a 32 × 32 matrix.
2. Plot a black dot on a white page if (*i*, *j*)-entry of the matrix is equal to 1, where 0 ≤ *i*, *j* < 32.

Though this scheme is quite primitive, it is effective for seeing the periodicity of binary data. Intuitively, it helps to find out the period by looking for a notable pattern such as a white line on the page.

### 3.2. Stochastic Process Test

In the above observation, one of the random number sequences can be regarded as a transition of bits whose output is yielded by referring to the previous several bits. Then, the authors consider a stochastic process, and the periodicity is verified by calculating its state transition. If the probability of the state transition is notable, then one can presume that the sequence has a certain periodicity and that the probability of appearances of bits may not be distributed uniformly.

The sequences used in this experiment are approximately 1M bits which are sampled by concatenating 1024 bits obtained from 1000 points. Then, each sequence is divided into 2 bits each, and the respective 2 bits are regarded as a state in a stochastic process. The probabilities of the state transitions are calculated. These processes are conducted with a single RO circuit, two RO circuits, and 50 RO circuits to compare the difference of distribution. It is noted that since the difference between these generators is the number of RO circuits, which provides the input for the XOR gate, the authors think it helps to reveal the effect of the XOR gate on the sequences.

## 4. Experimental Results and Considerations

This section explains several experimental results from the viewpoint of visualization and using the stochastic process. Additionally, considerations regarding these perspectives are also introduced in the latter part of this section.

### 4.1. Observation by Visualization

**Figure 5:**
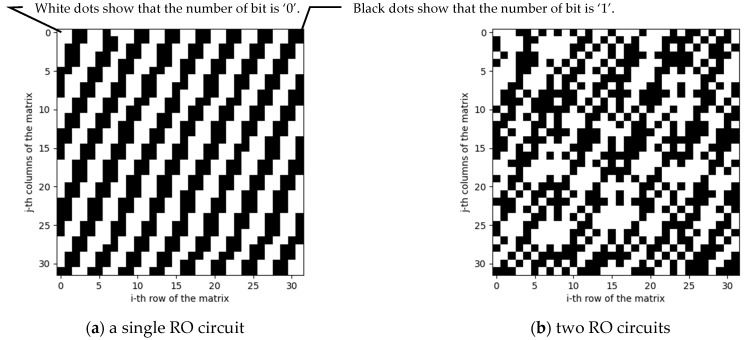
32 × 32 matrix data of the random number sequence.

Figure 5 shows the graphs that are obtained by following the steps in Section 3.1. The graphs of the single RO and two circuits are shown in Figure 5a,b, respectively. As seen from the figures, the output of a single RO has a regularity. On the other hand, it is found in Figure 5b that using two ROs with an XOR gate allows us to make the regularity noisy.

In other words, the authors can confirm that the XOR gate surely contributes to mixing the output from the two RO circuits, and it extends the period of oscillations.

### 4.2. Observation by Using Stochastic Process

**Figure 6:**
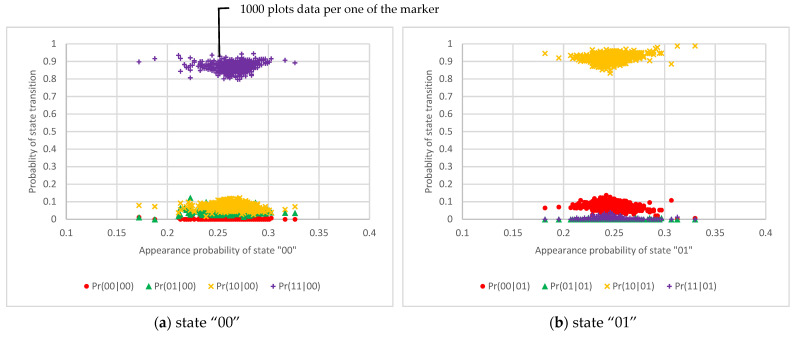

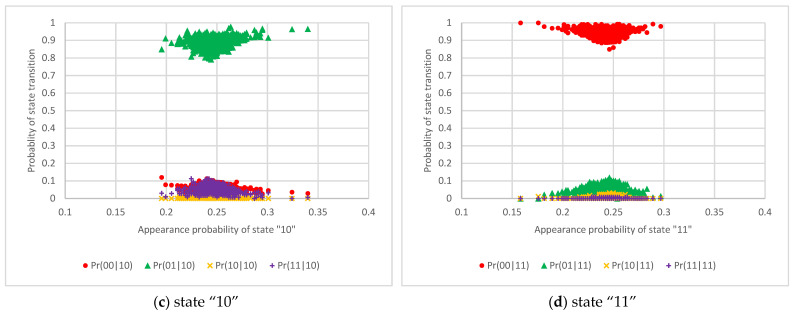
The scatter diagram of the 2-bit state transition probability from each state using one RO circuit.

**Figure 7:**
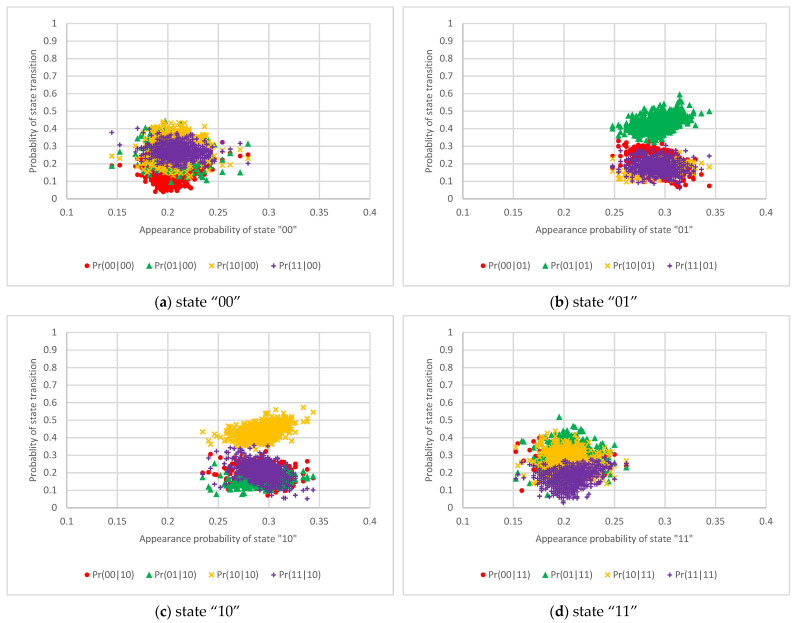
The scatter diagram of the 2-bit state transition probability from each state using two RO circuits.

**Figure 8:**
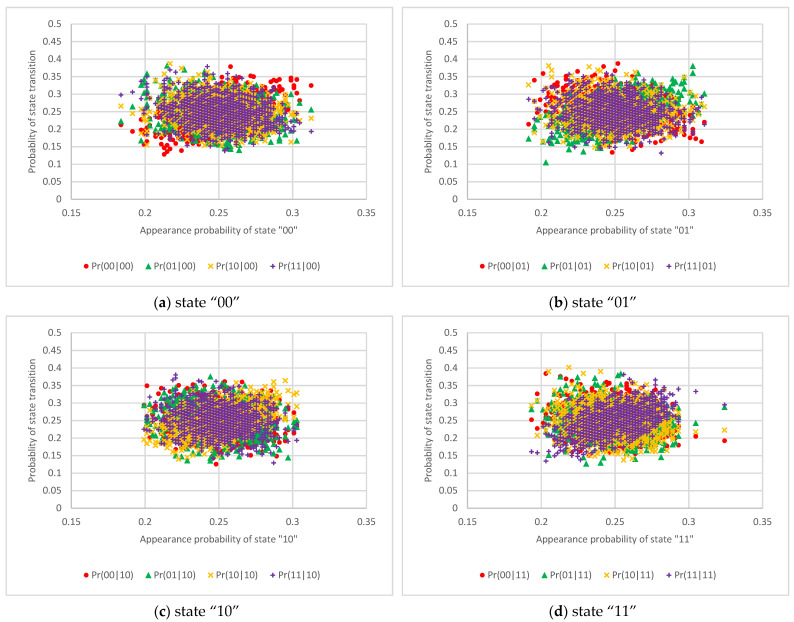
The scatter diagram of the 2-bit state transition probability from each state using 50 RO circuits.

The results of the state transition probabilities for one RO circuit, two RO circuits, and 50 RO circuits are shown in Figure 6, Figure 7 and Figure 8, respectively. These graphs show that the horizontal axis is the appearance probability of the respective 2-bit state and that the vertical axis is the probability of the state transition. Figure 6 shows that the probabilities of state transitions are not distributed uniformly and that the output values are cyclical. On the other hand, Figure 7 exhibits that the probabilities of state transitions are more evenly distributed than the results in Figure 6. Furthermore, Figure 8 reveals that the transition probabilities from each state distribute evenly.

### 4.3. Considerations

**Figure 9:**
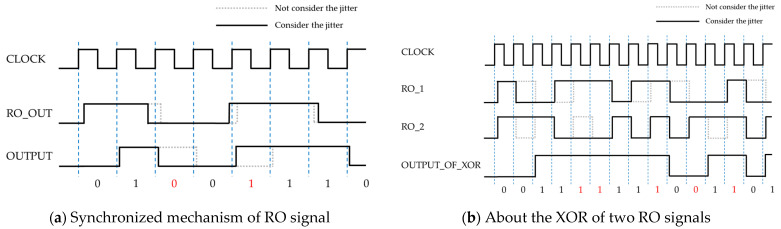
Example of the timing chart.

Let us begin by reviewing Figure 9a,b, which shows the timing chart of Figure 5a,b. In these figures, the continuous lines show the waveform that takes jitter into account, and the dashed lines show the ideal waveform, that is without jitter.

Figure 9a shows an OR circuit whose output is obtained by synchronizing the clock pulse. As one can see from the figure, a bit obtained from a positive edge of a certain clock can be affected by the jitter in terms of flipping. However, since the difference caused by the jitter is quite smaller than the clock width, it does not have enough effect to make the cycle of an RO irregular, as we confirmed from Figure 5a.

On the other hand, as seen from Figure 5b, a circuit having multiple ROs and an XOR enable us to make the output look more irregular than a circuit consisting of a single RO. This can be confirmed from the timing chart in Figure 9b.

Here, let us assume the use of an AND or an OR gate instead of an XOR gate. Since the reader can find that the appearance of 0’s and 1’s in the output of these gates is not distributed uniformly, one can easily conclude that these gates cannot contribute to generating bits uniformly under the above assumption.

Therefore, we think that the XOR gate in an RO circuit would be an essential factor for having a well-distributed sequence, and in what follows, we further consider the effect of an XOR gate for the cycle of a circuit having multiple ROs.

We assume that the periods of the two oscillation circuits are *t*_1_ and *t*_2_, respectively, and that the period of the output signal is *t*_0_. Then, the period of the output when the two oscillation circuits are combined by an XOR gate is expressed by the following equation:

*t*_0_ = l cm (*t*_1_, *t_2_*) (1)

If the periods *t*_1_ and *t*_2_ are equal, then *t*_0_ = *t*_1_ = *t*_2_. However, even if the circuit is designed to have the same period, the oscillation period of the RO circuit will have an error due to jitter. This is endowed by the fact that *t*_1_ and *t*_2_ are quite close to each other; however, they are not exactly equal. Thus, the greatest common divisor of this period is the high value. Thus, combining RO circuits with an XOR gate is said to contribute to making the oscillation period large, and as a result, the whole periods of output sequences become longer.

## 5. Conclusions

In this research, the authors focused on the construction of an RNG using RO circuits. It aimed to reveal the relationship between the periodicity of an RO circuit and the randomness endowed by introducing an XOR gate.

By taking into account the theoretical result by Sunar et al., the authors conducted two experiments from different viewpoints. According to the first experiment, it is found that the XOR gate contributes to making the periodic oscillation long and complex. In addition, the second experiment showed that each 2-bit state transition probability seemed to distribute evenly depending on the number of ROs. In other words, the XOR gate is expected to work to flatten the oscillation of the respective RO circuit.

Further investigations of this probability transition and its general cases are left for future works.

## Figures and Tables

**Table 1:** Experimental environment.

Environments for the Experiments	Conditions
The number of NOT gates in an RO	3
FPGA board	NEXYS A7 [15]
Frequency of internal clock	100 MHz
IDE	Vivado 2019.02
Programming language	SystemVerilog HDL, Verilog HDL

## Data Availability

Not applicable.
